# Supplementary material for: Analysis of the Bacterial Community and Fatty Acid Composition in the Bacteriome of the Lac Insect Llaveia axin axin
Source: Microorganisms. 2025 Aug 18;13(8):1930. doi: 10.3390/microorganisms13081930 (PMC12388429; doi:10.3390/microorganisms13081930)
Supplement: Supplementary file 1 [file microorganisms-13-01930-s001.zip › microorganisms-3777269-supplementary.pdf]

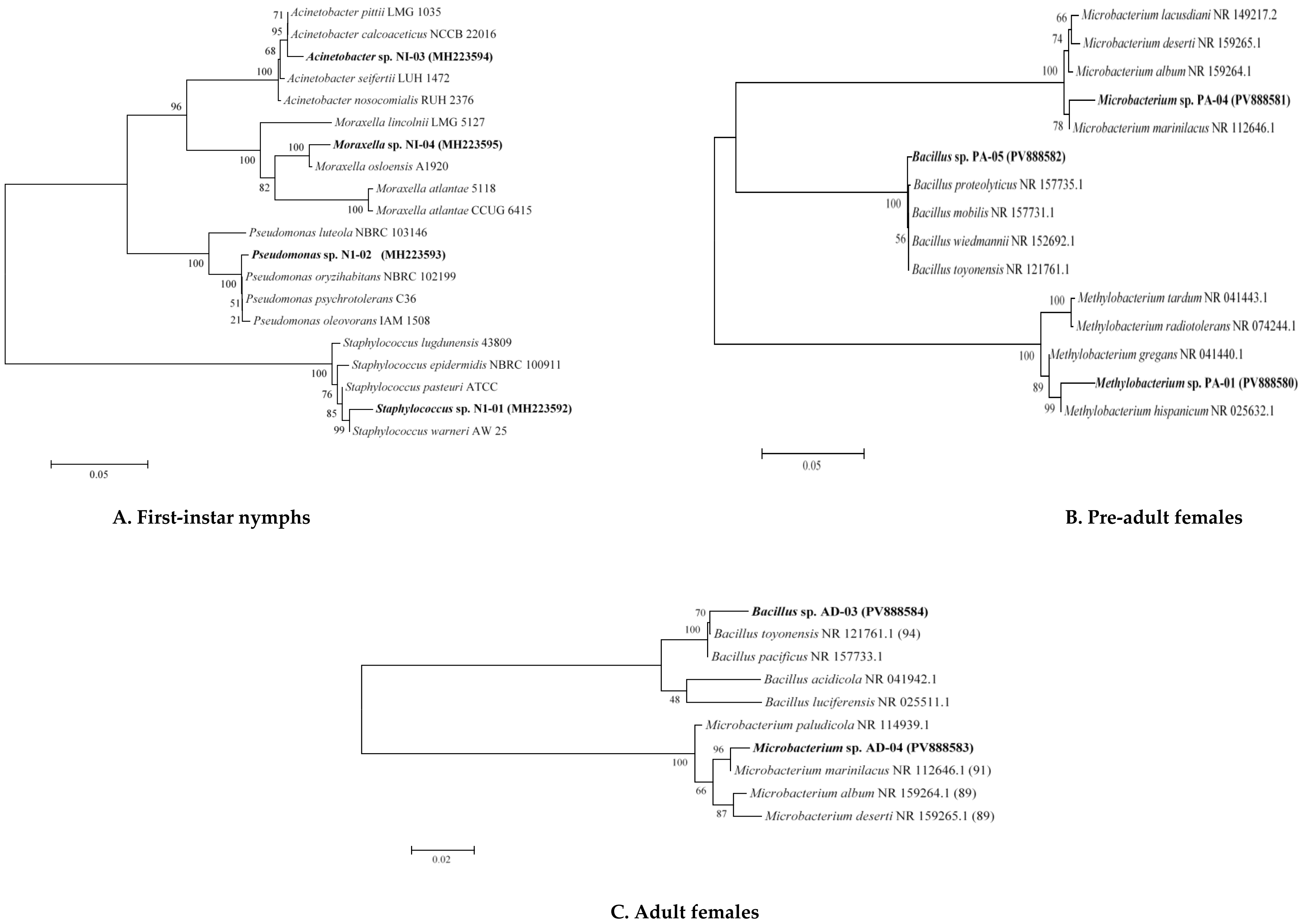

Figure S1. Neighbor-Joining phylogenetic trees based on 16S *r*RNA gene sequences from bacterial strains isolated from *Llaveia axin axin*. Strains identified in this study are shown in bold, with GenBank accession numbers in parentheses. **(a) First-instar nymphs**; (**b) pre-adult females; (c) adult females.**
